# Awake intubation for thoracic aortic aneurysm causing esophageal stenosis with food residues and compression of the pulmonary artery and left bronchi: a case report

**DOI:** 10.1186/s40981-022-00534-3

**Published:** 2022-06-21

**Authors:** Yasuhiro Suda, Ami Sugawara, Megumi Kanao-Kanda, Tomonori Shirasaka, Hiroyuki Kamiya, Hirotsugu Kanda

**Affiliations:** 1 Department of Anesthesiology and Critical Care Medicine, Asahikawa Medical University, Midorigaoka-higashi 2-1-1-1, Asahikawa, Hokkaido, 078-8510 Japan; 2 Department of Cardiac Surgery, Asahikawa Medical University, Midorigaoka-higashi 2-1-1-1, Asahikawa, Hokkaido, 078-8510 Japan

**Keywords:** Thoracic aortic aneurysms, Awake intubation, Superior laryngeal nerve block, Transesophageal echocardiography

## Abstract

**Background:**

Anesthetic management of thoracic aortic aneurysms (TAAs) is sometimes difficult due to fatal complications, including hypovolemic shock secondary to aneurysm rupture. We report the successful management of an impending rupture of a TAA with associated esophageal stenosis and compression of the pulmonary artery and left bronchi.

**Case presentation:**

An 83-year-old woman, diagnosed with an impending rupture of the ascending TAA, was scheduled to undergo emergency total aortic arch replacement. Computed tomography showed esophageal stenosis with significant amounts of food residues in the thoracic esophagus and compression of the pulmonary artery and bronchi. We performed awake intubation and superior laryngeal nerve block with light sedation to prevent aspiration and aneurysmal rupture, respectively. General anesthesia was induced immediately after the intubation. No intraoperative complications occurred.

**Conclusions:**

Performing awake intubation with a superior laryngeal nerve block and sedation may prevent aspiration of food residues and hemodynamic changes that may lead to rupture.

## Background

Anesthetic management of thoracic aortic aneurysms (TAAs) is sometimes difficult because of its fatal complications, such as hypovolemic shock due to an aneurysm rupture [1]. TAAs reportedly induce airway compression due to hypoxia and dysphagia. However, there are few reports on the anesthetic management of an impending rupture of TAA with both esophageal stenosis and airway compression. We report the successful management of an impending rupture of a TAA with associated esophageal stenosis as well as compression of the pulmonary artery and left bronchi.

## Case presentation

Written informed consent was obtained from the patient for the publication of this case.

**Fig. 1:**
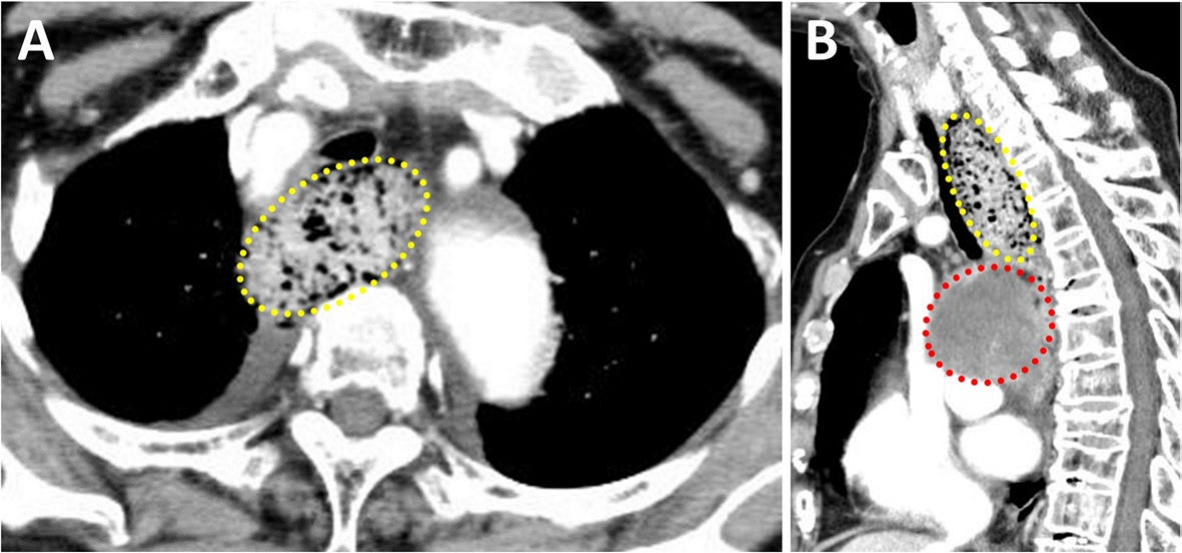
Axial section (**A**) and sagittal section (**B**) of preoperative thoracic computed tomography (CT). There is a significant amount of food residues in the upper thoracic esophagus (yellow dotted line circle) (**A**, **B**). Thoracic aortic aneurysm with impending rupture (red dotted line circle) is compressing the thoracic esophagus with food residues

**Fig. 2:**
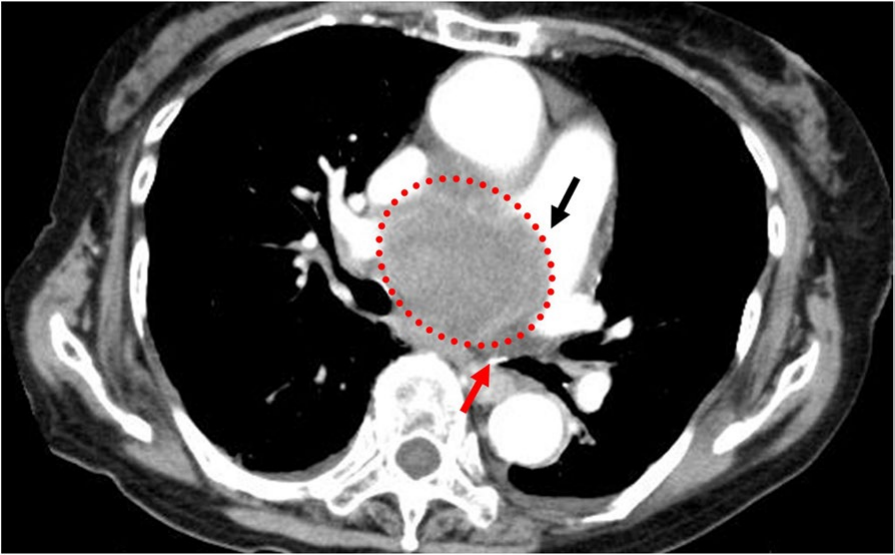
Preoperative CT scan showing a large thoracic aortic aneurysm (TAA) (red dotted line circle) impending rupture. TAA is compressing the pulmonary artery (black arrow) and left bronchi (red arrow)

An 83-year-old woman (height, 150 cm; weight, 58 kg), diagnosed with an impending rupture of an ascending TAA, was scheduled to undergo emergency total aortic arch replacement. She had a meal 2 h before the onset of chest pain and dyspnea, occurring 5 h prior to anesthetic induction. Due to the emergency nature of the case, the preoperative examination was not performed completely. Since no cardiac function tests were performed, details of preoperative cardiac function were unknown. The patient's medical history was unremarkable, with no history of medication use. Regarding preoperative blood test results, there was a slight increase in C-reactive protein (0.38 mg/dl) and fibrinogen degradation product (40.2 μg/ml) levels. Moreover, D-dimer levels (24.4 μg/ml) were elevated; however, the levels were not sufficient to cause disseminated intravascular coagulation. In the emergency department, computed tomography (CT) revealed an impending rupture of the ascending TAA and esophageal stenosis with significant amounts of food residues in the upper thoracic esophagus (Fig. 1). In addition, the TAA compressed the pulmonary artery and left bronchi (Fig. 2). To avoid TAA rupture and hypoxia due to cessation of respiration, the patient was intubated while being awake, without the insertion of a transesophageal echocardiography (TEE) probe. In the operating room, the patient was placed on a standard monitor. The electrocardiogram showed a sinus rhythm, and the heart rate was 105 beats per minute. The arterial blood pressure of the cannulated left radial artery had a systolic pressure of approximately 100 mmHg and a diastolic pressure of approximately 50 mmHg. The peripheral oxygen saturation was approximately 80% with oxygenation at 10 L/min via a reservoir-bag mask. After attaching the monitors, preoxygenation was administered with an FiO_2_ of 1.0 and oxygen flow rate of 10 L/min. This was sustained until the end of anesthesia induction.

A bilateral superior laryngeal nerve block was performed using an anatomical landmark-guided technique with 2 mL of 1% lidocaine [2]. We achieved this block using the greater horn of the hyoid bone and the superior horn of the thyroid cartilage as landmarks. Using a 27-gauge needle, we used the space through the thyroid membrane between them at a depth of 1 cm to inject lidocaine on both sides. Then, topical anesthesia was applied to the oropharyngeal mucosa with a 4% lidocaine spray. Awake endotracheal intubation was performed using a McGrath MAC® video laryngoscope with light sedation, induced by intravenous midazolam 3 mg and fentanyl 200 mg. After confirming the successful intubation, 50 mg of rocuronium and 2 mg of nicardipine were intravenously administered. During intubation, the pharyngeal reflex was mild and there was no aspiration, development of hypoxia, or rapid hemodynamic changes. The general anesthesia was maintained with a continuous infusion of remifentanil (0.2–0.3 mcg/kg/min), propofol (controlled target blood concentration at 1.0–2.0 mcg/mL, monitored by a bispectral index of 40–60), and single-dose infusion of rocuronium (10 mg per 30 min). Postoperatively, a single dose of fentanyl (600 mcg), a continuous infusion of dexmedetomidine (0.4 mcg/kg/h), and propofol (20 mg/h) were administrated intravenously.

There were no intraoperative complications. The patient was transferred to the intensive care unit. The operating time was 5 h 11 min, and the anesthetic time was 6 h 31 min. A total of 6194 mL of blood was lost, while 12,550 mL of blood was transfused. There were no complications, such as pulmonary aspiration, development of hypoxia, or TAA rupture. TEE was not performed. Postoperatively, the patient’s saturation in oxygenation improved greatly and the hemodynamic parameters were within normal ranges. The patient was extubated on postoperative day 5. The duration of mechanical ventilation was 116 h.

## Discussions

This is a case of an impending TAA rupture, complicated by esophageal stenosis with a significant amount of food residues, and hypoxia due to compression of the pulmonary artery and left bronchi. This patient had a high risk of aspiration because fasting had not been observed, and she developed esophageal stenosis resulting from residual food particles [3]. Moreover, the patient experienced hypoxia before anesthetic induction. During anesthesia induction, pulmonary aspiration, development of hypoxemia, and rapid hemodynamic changes that may lead to rupture were avoided. To achieve this, endotracheal intubation with a superior laryngeal nerve block was performed while the patient was awake under light sedation.

The common options for anesthesia induction in patients at risk of pulmonary aspiration include awake endotracheal intubation, and rapid sequence induction and intubation (RSII). During awake intubation, the patient can spit out the aspirated contents. Furthermore, awake intubation with oxygenation preserves spontaneous breathing and prevents hypoxia development [4]. In contrast, RSII can possibly increase the morbidity and mortality of patients with hypoxia [5–7]. Thus, awake intubation was performed in this patient.

Although the patient came to the hospital within 3 h from the onset of her symptoms, CT revealed signs of an impending rupture. This implied the rapid enlargement of the TAA and its near rupture. Therefore, the risk of rupture was considerably high. To prevent hemodynamic changes that may lead to rupture during intubation, light sedation was performed with intravenous administration of midazolam and fentanyl. A superior laryngeal nerve block was also conducted, and antihypertensives were administered. Previous case reports have documented successful awake intubation procedures with the use of a superior laryngeal nerve block [8]. The superior laryngeal nerve, a branch of the vagal nerve, provides sensory input from the lower pharynx and upper larynx, including the glottic surface of the epiglottis and the aryepiglottic folds [2]. The block decreases sensory inputs from the lower pharynx and upper larynx. This may contribute to the suppression of noxious stimuli that may cause rupture during intubation. However, there is an increased risk of aspiration because the block inhibits the deglutition reflex. This is circumvented by performing awake intubation, wherein the patient can spit out aspirated contents.

According to the American Society of Echocardiography and the Society of Cardiovascular Anesthesiologists guidelines [9], intubation was absolutely contraindicated in the present case due to the possibility of esophageal perforation secondary to esophageal stenosis. Esophageal stenosis due to TAA was observed in this patient. Thus, performing TEE would have caused esophageal perforation. Since the TAA with an impending rupture was located at the esophageal border, the TEE insertion could have caused TAA rupture.

In summary, this was a case of an impending TAA rupture, causing esophageal stenosis and hypoxia due to compression of the pulmonary artery and left bronchi. Performing awake endotracheal intubation with a superior laryngeal nerve block and light sedation effectively prevented food residue aspiration, hypoxia development, and hemodynamic changes that could have led to rupture in the present case. TEE probes should not be inserted in esophageal stenosis cases due to giant TAAs.

## Data Availability

Not applicable.

## Abbreviations

TAA: Thoracic aortic aneurysm
TEE: Transesophageal echocardiography
CT: Computed tomography
RSII: Rapid sequence induction and intubation
